# CsTFL1 inhibits determinate growth and terminal flower formation through interaction with CsNOT2a in cucumber

**DOI:** 10.1242/dev.180166

**Published:** 2019-07-29

**Authors:** Changlong Wen, Wensheng Zhao, Weilun Liu, Luming Yang, Yuhui Wang, Xingwang Liu, Yong Xu, Huazhong Ren, Yangdong Guo, Cong Li, Jigang Li, Yiqun Weng, Xiaolan Zhang

**Affiliations:** 1 Beijing Vegetable Research Center (BVRC), Beijing Academy of Agricultural and Forestry Sciences, Beijing Key Laboratory of Vegetable Germplasms Improvement, National Engineering Research Center for Vegetables, Beijing 100097, China; 2 Department of Horticulture, University of Wisconsin-Madison, Madison, WI 53706, USA; 3 Department of Vegetable Sciences, Beijing Key Laboratory of Growth and Developmental Regulation for Protected Vegetable Crops, China Agricultural University, Beijing 100193, China; 4 State Key Laboratory of Plant Physiology and Biochemistry, College of Biological Sciences, China Agricultural University, Beijing 100193, China; 5 USDA-ARS, Vegetable Crops Research Unit, 1575 Linden Drive, Madison, WI 53706, USA

**Keywords:** Cucumber, *CsTFL1*, Determinate growth, Fine mapping, CsNOT2a, Functional characterizations

## Abstract

Cucumber (*Cucumis sativus* L.) is an important vegetable crop that carries on vegetative growth and reproductive growth simultaneously. Indeterminate growth is favourable for fresh market under protected environments, whereas determinate growth is preferred for pickling cucumber in the once-over mechanical harvest system. The genetic basis of determinacy is largely unknown in cucumber. In this study, map-based cloning of the *de* locus showed that the determinate growth habit is caused by a non-synonymous SNP in *CsTFL1*. *CsTFL1* is expressed in the subapical regions of the shoot apical meristem, lateral meristem and young stems. Ectopic expression of *CsTFL1* rescued the terminal flower phenotype in the *Arabidopsis tfl1-11* mutant and delayed flowering in wild-type *Arabidopsis*. Knockdown of *CsTFL1* resulted in determinate growth and formation of terminal flowers in cucumber. Biochemical analyses indicated that CsTFL1 interacts with a homolog of the miRNA biogenesis gene CsNOT2a; CsNOT2a interacts with FDP. Cucumber CsFT directly interacts with CsNOT2a and CsFD, and CsFD interacts with two 14-3-3 proteins. These data suggest that CsTFL1 competes with CsFT for interaction with CsNOT2a-CsFDP to inhibit determinate growth and terminal flower formation in cucumber.

## Abstract (precis)

**Summary:** A novel insight into the molecular mechanisms of determinate growth in cucumber and a strategy for fine-tuning plant architecture using *CsTFL1* to adapt to different cucumber production systems.

## INTRODUCTION

The plant life cycle is highlighted with different developmental phases. During the vegetative phase in *Arabidopsis*, the primary shoot meristem generates rosette leaf primordia on its flanks. After integration of environmental signals and endogenous cues, the shoot apical meristem (SAM) converts into the inflorescence meristem (IM) that produces a few cauline leaves or bracts during the first inflorescence phase (I1), and then produces determinate floral meristems (FMs) from the periphery of the meristem during the second inflorescence phase (I2) (Ratcliffe et al., 1998). Two types of inflorescence architecture are found in flowering plants: indeterminate and determinate. In indeterminate plants, the main axis grows indefinitely and only produces flowers on its flanks. In plants with determinate inflorescence, the main axis is terminated and the shoot apical meristem converts into a flower.

Transition from vegetative growth to reproductive growth is a finely tuned process that is regulated by multiple genetic pathways, including photoperiod, vernalization, gibberellin, age and autonomous pathways (Amasino and Michaels, 2010; Boss et al., 2004; Jack, 2004; Komeda, 2004). In *Arabidopsis*, *TERMINAL FLOWER 1* (*TFL1*) and *FLOWERING LOCUS T* (*FT*) are key integrators of the floral transition pathways but act in an antagonistic manner. TFL1 is a repressor and FT is an activator for flowering transition (Baumann et al., 2015; Ratcliffe et al., 1998; Shannon and Meeks-Wagner, 1991). *TFL1* and *FT* belongs to the same phosphatidyl ethanolamine-binding proteins (PEBPs) family in *Arabidopsis* (Wickland and Hanzawa, 2015). A single amino acid change of Y88H in TFL1 and the relevant H85Y in FT are sufficient to convert the opposite functions of FT and TFL1 in flowering (Hanzawa et al., 2005). In addition, a divergent external loop of FT and TFL1 also confers their antagonistic activity on floral regulation (Ahn et al., 2006). *TFL1* is expressed in the central region of the shoot apical meristem, while the TFL1 protein is a mobile signal that is uniformly distributed in the whole meristem to repress flowering and maintain the indeterminate growth (Blazquez et al., 2006; Bradley et al., 1997; Conti and Bradley, 2007; Ratcliffe et al., 1999; Simon et al., 1996). The *Arabidopsis tfl1* mutants flower earlier than the wild type and display determinate growth with formation of a terminal flower (Alvarez et al., 1992; Bradley et al., 1997; Shannon and Meeks-Wagner, 1991). Overexpression of *TFL1* delayed the flowering time and extended both the vegetative and reproductive growth of *Arabidopsis* (Ratcliffe et al., 1998). Similarly, mutation of the *TFL1* homologous gene *CENTRORADIALIS* (*CEN*) in Antirrhinum caused conversion of indeterminate inflorescence to a terminal flower (Bradley et al., 1996). In tomato, the *TFL1* homolog *SELF-PRUNING* (*SP*) regulates the vegetative to reproductive switch in sympodial meristems (Pnueli et al., 1998). Upon constitutive overexpression of *RCN1* and *RCN2*, the *TFL1* homologs in rice, transition to reproductive phase was delayed, and the overexpression plant exhibited a more branched and denser panicle phenotype (Nakagawa et al., 2002). In maize, ectopic expression of the TFL1-like genes delayed flowering and altered inflorescence architecture by maintaining the meristem indeterminacy (Danilevskaya et al., 2010).

In *Arabidopsis*, the fate of the floral meristem is specified primarily by the meristem identity genes *APETALA1* (*AP1*), *LEAFY* (*LFY*) and *CAULIFLOWER* (*CAL*) (Benlloch et al., 2007; Gustafson-Brown et al., 1994; Weigel and Nilsson, 1995; Weigel et al., 1992)*.* FT interacts with the bZIP transcription factor FD and FD PARALOG (FDP) at the shoot apex to promote floral development through transcriptional activation of *LFY* and *AP1* (Abe et al., 2005; Wigge, 2006). On the other hand, TFL1 functions as a repressor for flowering, and thus inhibiting the expression of *LFY* and *AP1* in the subapical region of shoot apical meristem by competing with FT for interaction with FD and FDP (Benlloch et al., 2007; Hanano and Goto, 2011; Liljegren et al., 1999; Ratcliffe et al., 1999; Simon et al., 1996). Interestingly, *TFL1* can also be activated with the increased expression of *FT*, despite the antagonistic roles of *TFL1* and *FT* in controlling flowering (Jaeger et al., 2013). As a feedback, the expression of *TFL1* was suppressed by *AP1* but induced by *LFY* (Goslin et al., 2017). The same interaction patterns among *TFL1*, *FT* and *FD* were identified in rose and tomato (Randoux et al., 2014). In rice, the FT homolog Hd3a has been shown to interact with 14-3-3 proteins to form a complex, and then translocated to the nucleus where it binds to OsFD1, a homolog of *Arabidopsis* FD, but no direct interaction was detected between Hd3a and OsFD1 (Keicher et al., 2017; Taoka et al., 2011).

Cucumber (*Cucumis sativus* L.) is an important vegetable crop cultivated world-wide. Unlike the clear separation of the vegetative and reproductive phases, as observed in *Arabidopsis* and rice, cucumber displays vegetative growth and reproductive growth simultaneously: leaves are produced from the flank of the shoot apical meristem, while male or female flowers are produced from the axils of the leaf for the remaining growth period (Leonard, 1962). Determinacy is an important plant architecture trait in cucumber. Cucumber fruits are consumed fresh and immature. In many production systems, cucumber is produced under protected environments, and cucumber with indeterminate growth habit is favourable so the fruits can be harvested continuously for an extended growth period. In the USA, most cucumbers are grown in open fields and are harvested using the once-over machine harvest production system. In this system, cucumber with a determinate growth habit is preferable which is adapted to high-density planting and mechanical harvest (Denna, 1971; George, 1970; Hutchins, 1940; Miller and George, 1979; Pierce and Wehner, 1990).

Limited work has been carried out in cucumber to understand the genetic basis of determinate or indeterminate growth habit. Several studies indicated that the determinate growth in cucumber was controlled by a single recessive gene (Denna, 1971; Fazio et al., 2003; Sato et al., 2009). In the cucumber inbred line G421 (Gy7), the determinate growth habit was controlled by the *determinate* (*de*) locus that was located in cucumber chromosome 6 (Weng et al., 2010), but the gene(s) underlying the determinate growth or its regulatory network is unknown. In this study, we conducted map-based cloning of the *de* locus. We show that the determinate growth in G421 is due to a non-synonymous SNP in the *CsTFL1* (*Cucumis sativus TERMINAL FLOWER1*) gene. Further analyses indicated that CsTFL1 competes with CsFT for interaction with a homolog of the miRNA biogenesis gene *Negative on TATA less2* (*CsNOT2a*) to inhibit determinate growth and terminal flower formation in cucumber. Thus, our work suggests a strategy for fine-tuning plant architecture using CsTFL1 to adapt to different cucumber production systems.

## RESULTS

### A SNP in *CsTFL1* is responsible for the *determinate* (*de*) growth habit in G421 cucumber

Using 139 RILs from G421 (determinate)×H19 (indeterminate), we previously showed that the determinate growth habit in G421 cucumber was controlled by a single recessive gene *de* (Weng et al., 2010). In 2010 and 2011 field seasons, this RIL population and 946 F_2_ plants from the same crosses were observed for segregation for determinate growth habit. Among 139 RILs, 72 were indeterminate (*dede*) and 67 were determinate (*DeDe*) (χ^2^ test against 1:1, *P*=0.6715). Of the 946 F_2_ plants, 722 were indeterminate and 224 were determinate (χ^2^ test against 3:1, *P*=0.3480) (Tables S1 and S2). These data further confirm that a single recessive gene (*de*) underlies the determinate growth habit in G421.

**Fig. 1.**
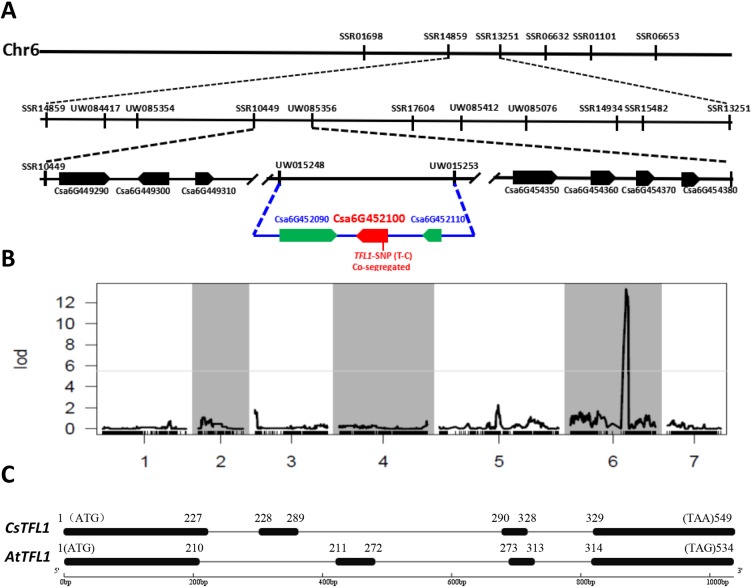
**Map-based cloning of the *determinate* (*de*) locus.** (A) Fine mapping with SSR markers using RIL-F_8_ and F_2_ plants delimited the *de* locus into an 18 kb region in chromosome 6 that contains three predicted genes, and a non-synonymous SNP was identified in the *Csa6G452100*/*CsTFL1*. (B) Genotyping by sequencing of 127 F_8_ RILs with SLAF-Seq identified 3563 markers that were used to located the *de* locus at the same region as above. (C) Gene structures of *CsTFL1* and *Arabidopsis AtTFL1.* Both contain four exons (black boxes) and 3 introns (grey lines). Numbers indicate the positions of exons.

Weng et al. placed the *de* locus onto chromosome 6 in a region between two markers, SSR14859 and SSR13251 (Weng et al., 2010). We identified 17 additional polymorphic SSRs in this interval. Linkage analysis in the RIL population allowed mapping of the *de* locus into a 493 kb region flanked by SSR10449 and UW085356 (Fig. 1A). Recombinants were screened with the two flanking markers in 946 F_2_ plants. Several cycles of fine mapping further narrowed down the *de* locus to an interval of 18 kb between UW015248 and UW015253, which harboured three predicted genes: *Csa6G452090* (for a purple acid phosphatase-20-like protein), *Csa6G452100* (*TFL1-like*) and *Csa6G452110* (for a germin-like protein). Sanger sequencing of the 18 kb regions in G421 and H19 revealed only one non-synonymous T (H19) to C (G421) polymorphism in the coding region of *Csa6G452100*. This SNP resulted in an amino acid substitution from S (serine) to P (proline) (Fig. 1A). A dCAPS marker (TFL1-SNP) was developed from this SNP for CAPS assay among 139 RIL and 946 F_2_ plants from the G421×H19 cross. This marker was co-segregating with the determinate/indeterminate growth habit among all plants examined.

In a separate effort, 127 F_8_ RILs were subjected to SLAF-Seq and a linkage map with 3563 SNPs was developed (Wei et al., 2014). This high-resolution map allowed fine mapping of *de* locus, which was placed in the same region as using SSR mapping method (Fig. 1B). These data suggested *Csa6G452100* to be the most possible candidate for the *de* locus.

*Csa6G452100* encodes a homolog of the *Arabidopsis TFL1* (*TERMINAL FLOWER1*) gene. As such, the cucumber homolog is hereafter referred to as *CsTFL1*. Cucumber *CsTFL1* was predicted to contain four exons and three introns, with a 549 bp coding region (CDS), which was structurally similar to the *TFL1* gene with 534 bp CDS in *Arabidopsis* (Fig. 1C).

### *CsTFL1* is a member of the PEBP gene family that is conserved among cucurbit crops

*CsTFL1* is a member of the PEBP gene family (Sato et al., 2009). BLAST search among three cucurbits (cucumber, melon and watermelon) draft genomes identified 21 PEBP family member genes with seven from each. A phylogenetic tree was constructed using protein sequences of the 21 cucurbit and six *Arabidopsis* PEBP members, which is shown in Fig. S1. The 27 PEBP members could be divided into four clades: the TFL1/CEN-like clade, the BFT-like clade, the FT/TSF-like clade and the MFT-like clade (Karlgren et al., 2011). Within each clade, the cucurbit PEBP members were clustered together to form a subclade that was distantly related to their *Arabidopsis* homologs. Interestingly, when compared with *Arabidopsis*, each cucurbit species had one fewer FT/TSF-like member, but one more TFL1/CEN-like or MFT-like member (Fig. S1).

We performed multiple sequence alignments of TFL1 proteins of G421 and H19 cucumbers with those from eight other species (Fig. S2). As expected, the key histidine amino acid residue that is essential for TFL1 function was conserved (red rectangle) (Hanzawa et al., 2005). However, variations were observed between cucurbit TFL1s and AtTFL1 in the segment B encoded by the fourth exon (Fig. S2), which has been shown to be essential for TFL1 activity (Ahn et al., 2006). The SNP in the determinate G421 cucumber caused S (serine) to P (proline) substitution. All TFL1s, including the TFL1 paralogous protein Csa3M776350 of cucumber had the same serine residue, except in G421 there was a proline residue at this position (blue rectangle), suggesting that this serine residue may play an essential role in regulation of shoot determinacy by TFL1. In addition, all cucurbit TFL1s could be distinguished from TFL1s of other species by a substitution of L (leucine) to F (phenylalanine). However, owing to several less-conserved residues, Csa3M776350 may have different functions from other cucurbit TFL1s.

It has been shown that the 5′ and 3′ regions of *Arabidopsis TFL1* play important regulatory roles in *TFL1* function (Serrano-Mislata et al., 2016). We aligned the genomic sequences, including their potential regulatory regions, (2.5 kb upstream and 1.5 kb downstream) of cucumber (*CsTFL1*), melon (*CmTFL1*), watermelon (*ClTFL1*) and *Arabidopsis* (*TFL1*) using mVISTA (Fig. S3A). Although the CDS of the *TFL1* genes displayed high similarity among *Arabidopsis* and cucurbits, the regulatory sequences were very different (Fig. S3A). Four conserved regulatory blocks (named A to D) were identified in *CsTFL1*, *CmTFL1* and *ClTFL1*, with two blocks in the 5′ region and two in the 3′ region. Using the plant MADS-box transcription factor-binding sites in the JASPAR database, we found six potential CArG-boxes in the 2 kb upstream of the 5′ end of *CsTFL1*, which was slightly higher than the average 4.4 predicted sites within the 2 kb upstream of all identified genes throughout the cucumber genome. Five potential CArG boxes were identified from the 2 kb downstream of the 3′ end of *CsTFL1*, which was twice the average of 2.5 predicted sites within the 2 kb downstream of all cucumber genes. Similarly, high numbers of CArG boxes were also predicted to be positioned in the 3′ regulatory region of the *AtTFL1* (Serrano-Mislata et al., 2016). Among the 11 CArG boxes predicted, six were conserved in the B, C and D regions (Fig. S3B).

### *CsTFL1* exhibits enriched expression in meristem and young stem of cucumber

**Fig. 2.**
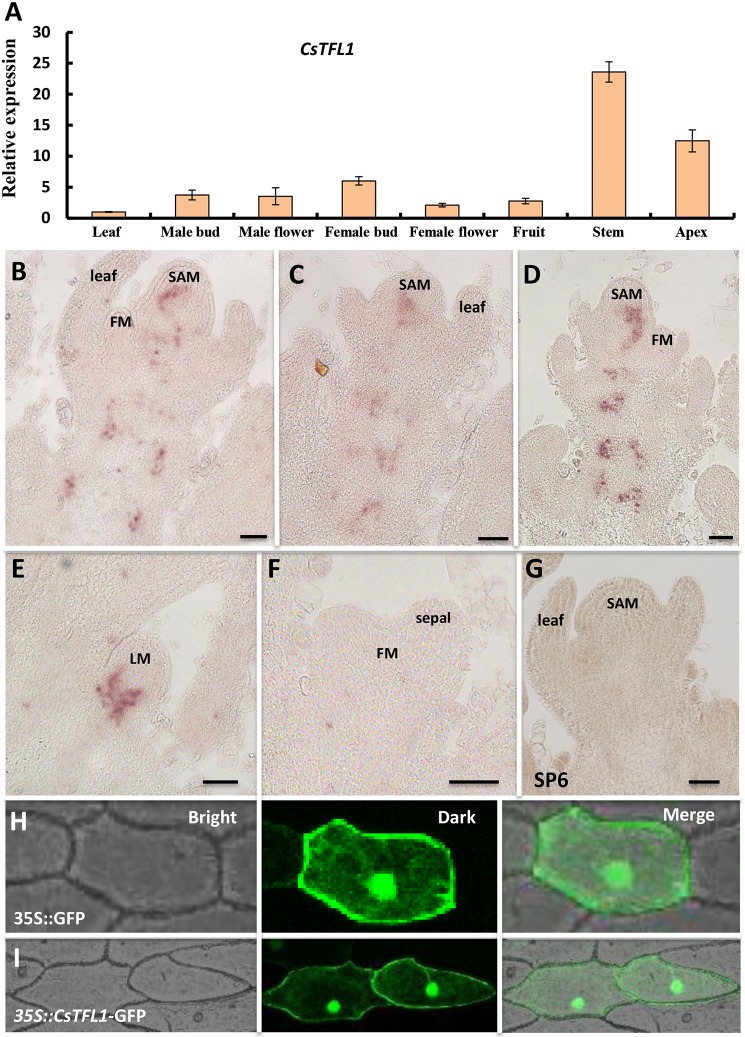
**Expression analysis of *CsTFL1* in cucumber.** (A) Relative expression of *CsTFL1* in different cucumber organs detected with qPCR. (B-F) *In situ* hybridization indicated that *CsTFL1* was expressed in the shoot apical meristem and stem (B-D), as well as in the lateral meristem (E), but not in the floral organ primordia (F). SAM, shoot apical meristem; FM, floral meristem; LM, lateral meristem. (G) The sense *CsTFL1* probe was hybridized as a control. (H,I) Subcellular localization showed that *CsTFL1* was found in both nucleus and cytoplasm. (I) GFP driven by the 35S promoter was used as a control. (H) GFP is shown in green. Data are mean±s.d. Scale bars: 50 μm.

To explore the expression pattern of *CsTFL1*, qRT-PCR was performed in cucumber tissues, including the leaf, male buds, opening male flowers, female buds, opening female flowers, fruit at anthesis, shoot apex after floral transition and young stem immediately beneath the 14-day-old shoot apex. The transcripts of *CsTFL1* were highly accumulated in stems, male buds, shoot apex and female buds (Fig. 2A). Next, *in situ* hybridization was applied to detect the spatial and temporal expression pattern of *CsTFL1*. Similar to that of *TFL1* (Bradley et al., 1997), *CsTFL1* was expressed in the centre region of shoot apical meristem after floral transition and in the discontinuous traces in the stem (Fig. 2B-D). *CsTFL1* was also expressed in the subapical region of the lateral meristem (Fig. 2E), but was absent in the floral meristem and floral organ primordia (Fig. 2F). No signal was detected upon hybridization with the sense *CsTFL1* probe (Fig. 2G). Subcellular localization of *CsTFL1* performed in onion indicated that *CsTFL1* was localized in both the nucleus and the cytoplasm (Fig. 2H,I).

### Ectopic expression of *CsTFL1* delays flowering in *Arabidopsis*

**Fig. 3.**
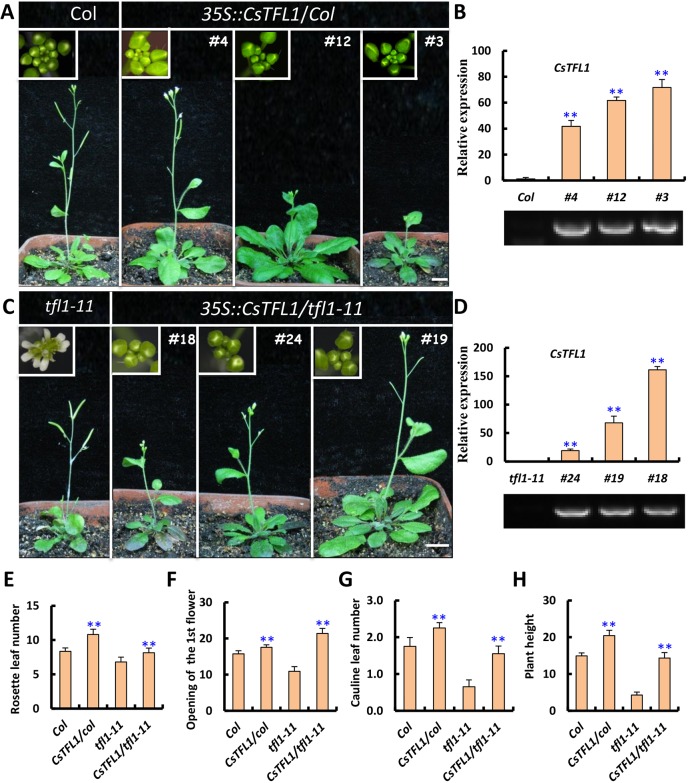
**Ectopic expression of *CsTFL1* in *Arabidopsis*.** (A-D) Phenotypic and qRT-PCR analysis of *CsTFL1* overexpression in wild-type *Arabidopsis* (A,B) and *tfl1-11* mutant (C,D) plants. Ectopic expression of *CsTFL1* delayed flowering in wild type and rescued the terminal flower phenotype of *tfl1-11* mutant. The insets in A and C show the magnified views of the corresponding inflorescence. (E-H) Quantification of the *CsTFL1* transgenic lines in *Arabidopsis*. (E) Rosette leaf number. (F) The days to the opening of the 1st flower. (G) Cauline leaf number. (H) Plant height. Data are mean±s.d. from 20 plants for each line. Asterisks indicate significant differences in transcript levels (Student *t*-test: **P<0.01) relative to the wild type or *tfl1-11* mutant. Scale bars: 2 cm.

To explore the putative function of *CsTFL1* in flowering regulation, we transformed *CsTFL1* driven by the cauliflower mosaic virus 35S promoter (CaMV 35S) into wild-type *Arabidopsis*. Twenty-two independent T1 transgenic lines were obtained. The flowering time of all transgenic lines was delayed by varying degrees. Three representative lines (mild line 4, medium line 12 and strong line 3) were chosen for further quantification. As shown in Fig. 3, the degree of delay correlated well with the expression level of *CsTFL1* (Fig. 3A,B). In the *35S::CsTFL1* transgenic lines, the number of rosette leaves was significantly increased (10.80 versus 8.35) (Fig. 3E), the days to the 1st flower opening were delayed (17.55 versus 15.75) (Fig. 3F) and the total number of cauline leaves was elevated (2.25 versus 1.75) (Fig. 3G). In addition, the mean vine length of transgenic plants was 5.55 cm longer than that in the wild type (Fig. 3H). Therefore, *CsTFL1* appears to delay flowering as well as to extend both vegetative growth and reproductive phases in *Arabidopsis*. However, the late flowering phenotype in *35S::CsTFL1* transgenic plants was not as dramatic as that of *35S::AtTFL1* transgenic plants in *Arabidopsis* (Ratcliffe et al., 1998).

We next transformed the *35S::CsTFL1* construct into the *Arabidopsis tfl1-11* mutant background. Twenty independent T1 transgenic lines were obtained. The *tfl1-11* mutant plants displayed early flowering and produced a terminal flower in *Arabidopsis* (Shannon and Meeks-Wagner, 1991). We found that ectopic *CsTFL1* expression can rescue the terminal flower phenotype of *tfl1-11* (Fig. 3C,D). Similarly, the numbers of rosette leaves, the days to the first flower opening, the total number of cauline leaves as well as plant height were largely recovered (Fig. 3E-H).

### Downregulation of *CsTFL1* leads to determinate growth in cucumber

**Fig. 4.**
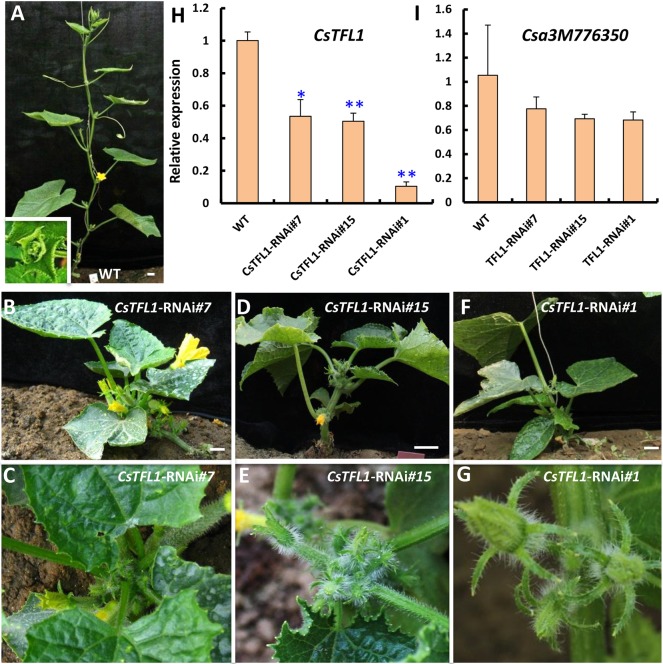
**Phenotypic characterization of *CsTFL1*-RNAi plants in cucumber.** (A) A wild-type cucumber plant transformed with empty vector and a normal shoot apex (inset). (B-G) Three representative *CsTFL1*-RNAi plants with significantly reduced plant height (B,D,F) and terminal flowers (C,E,G). (H,I) Expression analysis of *CsTFL1* (H) and its paralogous gene *Csa3M776350* (I) in *CsTFL1*-RNAi lines by qRT-PCR. Three biological replicates and three technical replicates were performed for each gene. Data are mean±s.d. Asterisks indicate significant differences relative to wild type (Student *t*-test: *P<0.05; **P<0.01). Scale bars: 2 cm.

To further characterize the function of *CsTFL1* in cucumber, a 35S promoter followed with a double-strand RNA interference (RNAi) construct containing the relatively specific region (1-216 bp coding sequence) of *CsTFL1* (*CsTFL1-RNAi*) was transformed into the indeterminate cucumber inbred line R1461. Six independent transgenic lines were obtained, and three representative lines (7, 15 and 1) were chosen for further analyses (Fig. 4). When compared with the wild-type plants transformed with empty vector, the height of transgenic plants was greatly reduced; all transgenic plants displayed obvious determinate growth with terminal flowers at the shoot tip (Fig. 4A-G). Consistently, the expression of *CsTFL1* in these transgenic lines was significantly reduced relative to wild type (Fig. 4H), whereas the expression of the *CsTFL1* paralogous gene *Csa3M776350* was unaffected (Fig. 4I), indicating that the knock down by RNAi is specific to *CsTFL1*. Owing to the severe phenotypes in *CsTFL1-RNAi* transgenic lines, we were unable to obtain seeds for further characterization.

### Protein interaction assays reveal possible CsTFL1-related regulatory network

**Fig. 5.**
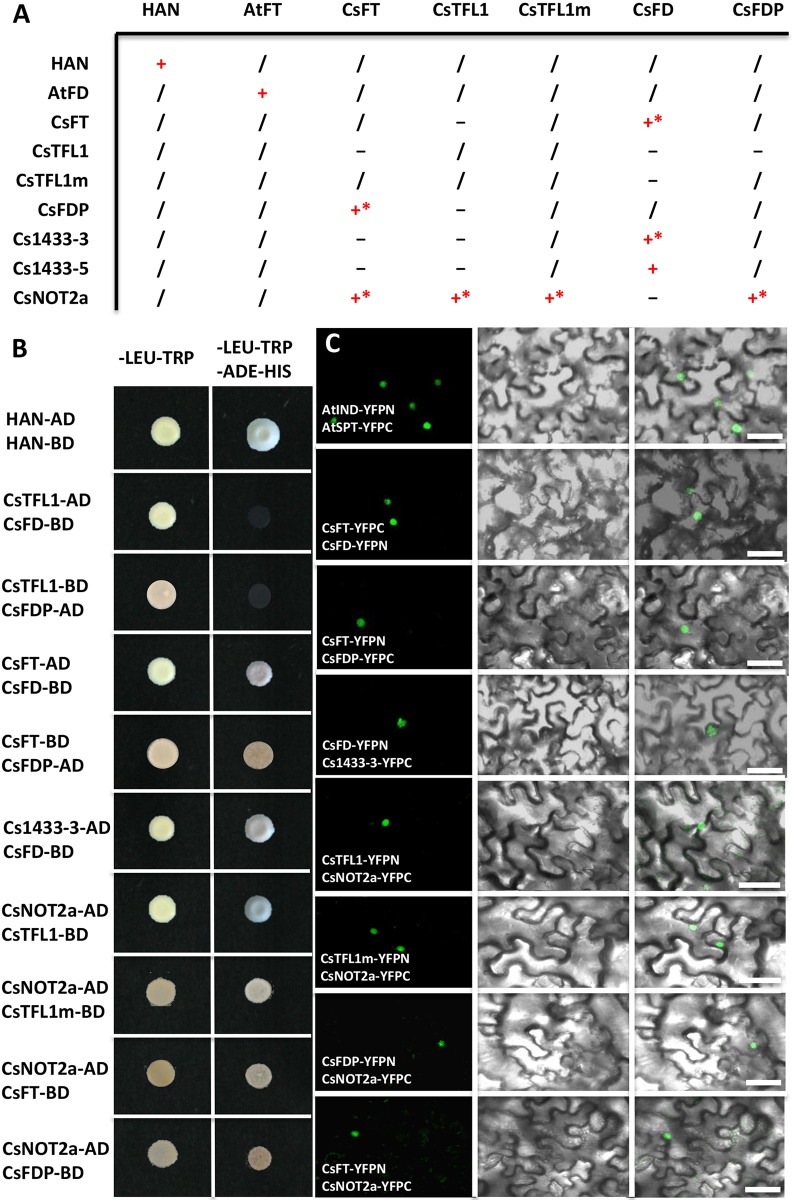
**Protein interactions detected with Y2H and BiFC assays.** (A) Summary of detected interactions in this study: +, positive interaction; *, interaction was confirmed by BiFC; −, no interaction; /, not applicable. (B) Y2H assay. CsTFL1, CsTFL1m (the amino acid serine was changed into proline), CsFT, CsFD, CsFDP, CsNOT2a and Cs14-3-3 proteins were fused to the GAL4 activation domain (AD) or DNA-binding domain (BD) to generate the bait constructs or prey constructs. The combination of AtHAN-BD and AtHAN-AD was used as the positive control (Zhang et al., 2013). (C) BiFC assay. A combination of INDEHISCENT (IND)-YFPC and SPATULA (SPT)-YFPN were used as positive controls (Girin et al., 2011). Dark-field, bright-field and merged channels are showed sequentially from left to right. Scale bars: 50 μm.

Previous studies have shown that both TFL1 and FT can interact with FD/FDP and 14-3-3, and function antagonistically to regulate downstream target genes in *Arabidopsis* (Hanano and Goto, 2011). However, in rice, OsFD1 displayed no direct interactions with Hd3a (FT homolog), but instead required the 14-3-3 proteins as the bridge to form a transcriptional complex (Keicher et al., 2017; Taoka et al., 2011). To explore the putative protein interactors of CsTFL1, a yeast two hybrid (Y2H) assay was performed. *HANABA TARANU (HAN)*, the boundary gene of meristem and organ primordia, was fused to the GAL4 DNA-binding domain (BD) and activation domain (AD) to serve as the positive control (Zhang et al., 2013). We found that CsFT interacts with CsFD and CsFDP, and CsFD interacts with two 14-3-3 proteins (14-3-3-3 and 14-3-3-5), but no direct interactions have been detected between CsFT and the two 14-3-3 proteins (Fig. 5A,B). Notably, no interactions were found between CsTFL1 or CsTFL1m (S71P) and CsFD, CsFDP or Cs14-3-3 proteins, or between CsTFL1 and CsFT (Fig. 5A,B, Fig. S4). These results suggested that the interaction patterns among *TFL1*, *FT* and *FD* in cucumber are different from that in *Arabidopsis*.

In order to identify the potential interacting proteins of CsTFL1, Y2H library screening was performed and CsTFL1 was served as the bait. The results showed that CsTFL1 directly interacts with the cucumber homolog of Negative on TATA less2 (CsNOT2a) (*Csa6G302150*) (Fig. 5A,B) (Collart and Struhl, 1994). NOT2 is the core member of the CARBON CATABOLITE REPRESSION4 (CCR4)-NOT complex, and is involved in miRNA biogenesis during male and female gametophyte development in *Arabidopsis* (Collart and Panasenko, 2012; Denis and Chen, 2003; Wang et al., 2013). Mutations in *NOT2a* or *NOT2b* result in aborted ovules and degenerated pollens in *Arabidopsis* (Wang et al., 2013). In addition, CsNOT2a was found to interact with CsFT and CsFDP, but not with CsFD (Fig. 5A,B, Fig. S4).

To verify the protein interactions *in vivo*, bimolecular fluorescence complementation (BiFC) assay was performed in the tobacco (*Nicotiana tabacum*) leaf. A combination of INDEHISCENT (IND)-YFPC and SPATULA (SPT)-YFPN was used as a positive control (Girin et al., 2011) (Fig. 5C). The fluorescent signals of YFP were observed in the cell nucleus upon co-infiltration of CsFT and CsFD, or CsFD and Cs14-3-3-3 (Fig. 5C), suggesting that CsFD acts as a linker between CsFT and Cs14-3-3 proteins in cucumber. No fluorescent signal was detected upon co-infiltratation of CsTFL1 and CsFD. Similarly, fluorescent signals were observed upon co-infiltration of CsNOT2a with CsTFL1, CsFT or CsFDP (Fig. 5C), indicating that CsNOT2a can physically interact with CsTFL1, CsFT and CsFDP in cucumber.

## DISCUSSION

### The amino acid residue S71 is essential for the function of *CsTFL1* in cucumber

In *Arabidopsis*, TFL1 is a repressor but FT is an activator for flowering regulation (Baumann et al., 2015; Ratcliffe et al., 1998; Shannon and Meeks-Wagner, 1991). A single amino acid change of Y88H in TFL1 and the relevant H85Y in FT is sufficient to reverse the functions of TFL1 and FT, respectively (Hanzawa et al., 2005). In tomato, the single amino acid change (P76L) was found to be pivotal for the ‘determinate’ phenotype in *sp* mutant (Pnueli et al., 1998). In the present study, we found that one non-synonymous T (H19) to C (G421) SNP in *CsTFL1*, which changed the conserved hydrophilic serine to hydrophobic proline, led to the determinate growth in G421 cucumber (Fig. 1A), suggesting that this serine residue plays an essential role in the ability of CsTFL1 to regulate the determinate growth in cucumber. In addition, the TFL1 proteins of cucumber, melon and watermelon displayed several conserved residues in the coding region, as well as four conserved regulatory blocks containing putative MADS-box transcriptional factor binding sites (CArG box) (Figs S2 and S3) (Liu et al., 2013), which may be important for TFL1 functioning in cucurbits.

### Opposite roles of CsTFL1 and CsFT in regulating determinate growth and terminal flowering in cucumber

**Fig. 6.**
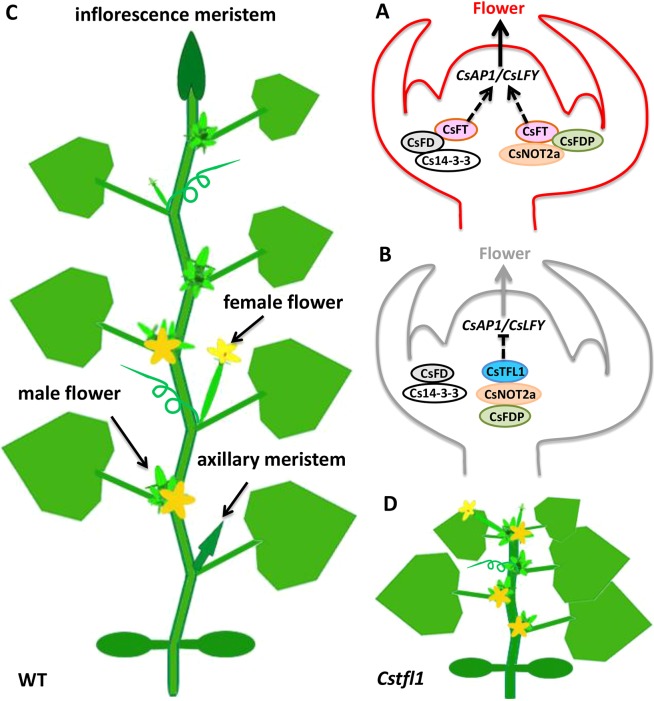
**Working model of CsTFL1 in regulating determinate growth in cucumber.** (A) CsFT promotes determinate growth and terminal flower formation in cucumber. CsFT can directly interact with CsFD or CsFDP, which may form a ternary complex with Cs14-3-3 or CsNOT2a to activate *CsAP1* and *CsLFY* expression in the shoot apical meristem of cucumber to regulate determinate growth. (B) CsTFL1 stimulates indeterminate growth and inhibits terminal flower formation in cucumber. CsTFL1 was unable to directly interact with CsFD or CsFDP. However, CsTFL1 may cooperate with CsFDP through the bridge protein CsNOT2a to inhibit the transcription of *CsAP1* and *CsLFY*. (C) A classical cucumber plant with indeterminate growth. The plant carries on vegetative growth and reproductive growth simultaneously. Leaves were produced from the flank of the shoot apical meristem, and unisexual flowers were produced from the leaf axils. (D) Disruption of the *CsTFL1* results in formation of terminal flowers and thus determinate growth in cucumber.

In *Arabidopsis*, both TFL1 and FT interact with FD/FDP and 14-3-3, and function antagonistically to regulate the downstream floral meristem identity genes, including *LFY* and *AP1* (Hanano and Goto, 2011). However, in rice, Hd3a (FT homolog) shows no direct interaction with OsFD1, but instead requires rice 14-3-3 protein as the bridge to form a transcriptional complex with OsFD1 (Keicher et al., 2017; Taoka et al., 2011). In tomato, there also are associations between SP (TFL1 homolog) and 14-3-3 isoforms, a NIMA-like kinase (SPAK) and a bZIP factor SPGB (Pnueli et al., 2001). Here, we found that CsFT can directly interact with CsFD and CsFDP, which may form a protein complex with Cs14-3-3 or CsNOT2a to activate *CsAP1* and *CsLFY* expression in the shoot apical meristem, and thus promote determinate growth and formation of terminal flowers in cucumber (Figs 5 and 6A). On the other hand, no interactions were detected between CsTFL1 and CsFD or CsFDP, or between CsTFL1 and the two Cs14-3-3 proteins, as evidenced from Y2H and BiFC assays (Fig. 5, Fig. S4). Instead, CsTFL1 was found to interact with CsNOT2a, which also interacts with CsFDP. Therefore, CsNOT2a may serve as a linker protein between CsTFL and CsFDP to form a complex that is antagonistic to the function of CsFT complex to inhibit the transcription of *CsAP1* and *CsLFY* (Figs 5 and 6B). The balance between CsTFL1 and CsFT specifies the indeterminate versus determinate growth in cucumber. Interestingly, the amino acid change at S71P did not disrupt the interaction between CsTFL and CsNOT2a in the Y2H assay (Fig. 5, Fig. S4). Considering that impairment of NOT2a leads to mislocalization of its direct interaction partner, DICERLIKE1 (DCL1), in *Arabidopsis* (Wang et al., 2013), the S71P change in CsTFL may affect the subcellular localization of its protein partners in cucumber, and or disruption of as yet unidentified interacting proteins.

### Understanding the regulatory mechanisms of determinate growth may facilitate manipulation of plant genetic architecture in cucumber production

The *TFL1* gene has been shown to regulate the inflorescence architecture and flowering time in plant species with a progressive mode (e.g. *Arabidopsis*) or an alternative mode (e.g. tomato) of vegetative growth and reproductive growth (Ratcliffe et al., 1998; Wickland and Hanzawa, 2015). In cucumber, except for the short juvenile stage, cucumber plants carry on vegetative growth and reproductive growth simultaneously. Leaves are generated from the periphery of the shoot apical meristem, and cucumber unisexual flowers are produced from the leaf axils (Fig. 6C). In the present study, we show that disruption of the function of *CsTFL1* by RNAi led to determinate growth and formation of terminal flowers in cucumber (Figs. 4 and 6D), suggesting the conserved role of TFL1 in regulating the transition from indeterminate to determinate growth habits in plants. Although ectopic expression of *CsTFL1* delayed flowering in wild-type *Arabidopsis*, no difference in flowering time was observed between wild-type R1461 and *CsTFL1-*RNAi transgenic plants. This suggests that additional players other than CsTFL1 may exist that can regulate flowering time in cucumber.

The determinate/indeterminate growth habit is an important plant architecture trait in cucumber and many other vegetable crops. In cucumber production under protected environments, indeterminate growth is ideal. However, often environmental stresses cause terminal flowering thus loss of cucumber productivity. In certain production systems, like the once-over machine harvest system in the USA, determinate cucumber may be advantageous (Fazio et al., 2003; Weng et al., 2010). Thus, results from this study allow us to have a better understanding of the mechanisms of CsTFL1 functioning. On the practical side, they also suggest a strategy for fine-tuning the indeterminate/determinate growth habit using different alleles of *CsTFL1* or by modulating *CsTFL1* expression levels to adapt to different cucumber production systems.

## MATERIALS AND METHODS

### Plant materials and growth conditions

Four cucumber inbred lines, G421, H19, R1461 and 9930, were used in this study. G421 and H19 were used for construction of the mapping populations for the *de* locus. H19 and G421 are typical American pickling cucumbers with short fruits. H19 was a monoecious and indeterminate line with small leaves, flowers and fruits but multiple branches (5 to 15). G421 was a gynoecious and determinate line, which possessed standard-sized leaves and few branches (1 to 3) (Yang et al., 2018). R1461 is a Northern China type cucumber and used for expression analysis and genetic transformation. The 9930 line with reference genome was used to construct the yeast two-hybrid library (Huang et al., 2009). Seeds of the Columbia (Col) *Arabidopsis* and the *tfl1-11* mutant (Hou and Yang, 2009) were obtained from the Arabidopsis Biological Resource Center (ABRC). The *Arabidopsis* plants were grown in a growth chamber under 16 h/8 h of light/dark at 22°C.

### Fine mapping of *determinate* (*de*) locus in cucumber

The determinate line G421 (*dede*) and the indeterminate line H19 (*DeDe*) were used to construct a RIL population (139 F_8_ lines) (Weng et al., 2010) and an F_2_ population (946 plants) for fine genetic mapping and cloning of the *de* gene. Phenotyping of all plants was performed in replicated field trials (for RILs) in the University of Wisconsin-Madison Hancock Agricultural Research Station in 2010 (RILs) and 2011 (RIL and F_2_ plants). The growth habit of each plant (determinate or indeterminate) was determined from 3-week-old seedlings until the end of the field season. A plant with the main stem ending in a flower cluster was classified as determinate, which also had shorter vine length when compared with the wild type (Weng et al., 2010). The published simple sequence repeats (SSRs) (Cavagnaro et al., 2010; Ren et al., 2009) were used to construct the genetic map for the *de* locus. For fine mapping, the two parental lines G421 and H19 were re-sequenced at 30× genome coverage using Illumina Hi-Seq 2000 for marker discovery. A linkage map was also developed based on genotyping-by-sequencing (GBS) of 127 F_8_ RILs using specific-locus amplified fragment sequencing (SLAF-Seq) as described by Wei et al. (2014). DNA exaction, PCR amplification of molecular markers and gel electrophoresis followed established protocols (Wen et al., 2015). Linkage analysis of the *de* locus with molecular markers in segregating populations was performed with JOINMAP 4.0 by using a minimum LOD threshold of 4.0 and the Kosambi mapping function.

### Identification of CsTFL1 homologs in cucurbits and phylogenetic analysis

The coding sequences (CDS) of *CsTFL1*, *TFL1* and *FT* genes were used in BLAST searches of their homologs in cucumber, melon and watermelon (www.cucurbitgenomics.org/). All 21 *TFL/FT* homologs identified from the three cucurbit species were verified in the Pfam protein family database (xfam.org/) (Bateman et al., 2004) to confirm their identity as the phosphatidyl ethanolamine-binding protein (PEBP) family members. Multiple sequence alignment was performed using Cluster W in DNAMAN (Version 7) or the GenomeNet online server (www.genome.jp/tools-bin/clustalw) with default settings. An unrooted phylogenetic tree was constructed for the 21 cucurbit PEBP protein sequences as well as for the six members from *Arabidopsis* using the neighbour-joining (NJ) method with MEGA6.0 software (bootstrap=1000) (Tamura et al., 2013). Gene information used in this study was listed in Table S3.

### Analysis of *TFL1 cis*-regulation regions in cucurbit crops

The *TFL1* coding region and its upstream 2.5 kb and downstream 2 kb sequences from cucumber, melon, watermelon and *Arabidopsis* were aligned using mVISTA (genome.lbl.gov/vista/index.shtml) (Frazer et al., 2004) (shuffle-LAGAN alignment, window size=100 bp, minimum sequence identity=50%). The 2 kb upstream and 2 kb downstream sequences of all identified genes in cucumber genomes were analysed using the R package Transcription Factor Binding Site (TFBS) Analysis and data package for JASPAR 2018 to predict the MADS transcription factor-binding region (CArG boxes) (Khan et al., 2018) (predicted binding site score>12; relative score>0.8).

### Candidate gene cloning, structure and expression analysis

Total RNA was extracted from the apex of the indeterminate cucumber line R1461 using a Quick RNA Isolation Kit (Waryoung) and cDNA was synthesized using the TianScript II RT Kit (Tiangen Biotech). Specific primers were used to obtain the full-length coding sequence of *CsTFL1* (XM_011659396.1). Gene structure analysis of *CsTFL1* was performed using the GSDS program (gsds.cbi.pku.edu.cn/). Primer information is listed in Table S4.

Total RNA was extracted from different cucumber tissues and *Arabidopsis* inflorescences using a Quick RNA Isolation Kit (Waryoung); cDNA was synthesized using the TianScript II RT Kit (Tiangen Biotech). Quantitative real-time RT-PCR (qRT-PCR) was performed with the ABI PRISM 7500 Real-Time PCR System (Applied Biosystems). The cucumber *Ubiquitin extension protein* (Csa000874) and *Arabidopsis ACTIN2* (AT3G18780) genes were used as internal references to normalize the expression data. Three biological and three technical replicates were performed in each qRT-PCR experiment. The primers used for qRT-PCR are listed in Table S4.

### *In situ* hybridization

Cucumber shoot apexes of 14-, 16- and 18-day-old seedlings were fixed in 3.7% formol-acetic-alcohol (FAA) and *in situ* hybridization was performed as described previously (Zhang et al., 2013). Primers targeted for the unique region of *CsTFL1* (1-216 bp) were used for PCR amplification to synthesize the sense and antisense probes by SP6 and T7 polymerase, respectively. The primer information is listed in Table S4.

### Subcellular localization

The *CsTFL1*-coding sequence without the stop codon was fused with the GFP-coding sequence, and then inserted into the plasmid pUC-SPYNE through SpeI and SmaI cleavage sites. Subcellular localization was performed in onion (*Allium cepa*) epidermal cells according to the established protocol (Girin et al., 2011). Images were taken by a confocal laser-scanning microscope (Carl Zeiss LSM 510, Germany) excited at a 488 nm wavelength. Relevant primer information is listed in Table S4.

### Ectopic expression of *CsTFL1* in *Arabidopsis*

The full-length *CsTFL1*-coding sequence was amplified and inserted into the pBI121 vector through XbaI and XmaI cleavage sites to make the *CsTFL1* overexpression construct. The resultant construct was transferred into *Agrobacterium* by electroporation and then transformed into Col (WT) and *tfl1-11* mutant plants through the floral dip method (Clough and Bent, 1998). The transgenic plants were screened on the Murashige and Skoog (MS) medium containing 40 mg l^−1^ kanamycin. The primers used for vector construction are listed in Table S4.

### Cucumber transformation

To generate RNAi constructs, the 220 bp sense and antisense fragments from the 5′ end of *CsTFL1* were amplified using gene-specific primers containing SpeI (5′ end)/BamHI (3′ end) and AscI (5′ end)/SwaI (3′ end) restriction sites, respectively. The two fragments were inserted into the PFGC-1008 vector. The empty PFGC-1008 vector was used as the control. The recombinant *CsTFL1*-RNAi construct and the empty PFGC-1008 vector were introduced into *Agrobacterium* and then transformed into cucumber inbred line R1461 as previously described (Ding et al., 2015a). For *CsTFL1*-RNAi transgenic line, the 2-day-old cotyledons were incubated in MS liquid medium with *Agrobacterium* harbouring the recombinant *CsTFL1*-RNAi construct for 12 min and placed on MS solid medium with 0.5 mg l^−1^ 6-BA and 1 mg l^−1^ABA. Upon culturing for 2 days in the dark, the cotyledons were transferred to MS selected medium with 100 mg l^−1^ chloromycetin. After 2-3 weeks, the differentiated shoots from cotyledons were cut and transferred to the MS rooting medium to generate the *CsTFL1*-RNAi transgenic lines. The primers used for vector construction are listed in Table S4.

### Yeast two hybrid library screening

The coding sequence of *CsTFL1* was cloned into the pGBKT7 to construct the bait vector BD-CsTFL1. A normalized cucumber Mate & Plate library was constructed by Shanghai OE Biotech using equal amounts of cDNA obtained from leaf, shoot tip and flowers of the cucumber inbred line 9930. The bait BD-CsTFL1 was used to screen the Mate & Plate library according the Matchmaker Gold Yeast Two-Hybrid System User Manual (Clontech). The full-length coding sequence of the identified interacting protein was cloned into the pGADT7. The resultant pGADT7 vector and pGBKT7 or BD-CsTFL1 were co-transformed into the Y2HGOLD yeast strain and selected on SD--Ade/-His/-Leu/-Trp to verify the interaction. In addition, full-length coding sequences for *CsTFL1*, *CsTFL1m*, *CsFT*, *CsFD*, *CsFDP*, *Cs14-3-3-3* and *Cs14-3-3-5* were cloned into pGADT7 (prey) or pGBKT7 (bait) vectors, sequenced and then transformed into the yeast strain AH109. The bait and prey vectors were transformed following the instructions of Matchmaker GAL4 Two-Hybrid System 3 & Libraries (Clontech). The protein interaction assay followed the methods of Ding et al. (2015b). The primer information is listed in Table S4.

### Bimolecular fluorescence complementation (BiFC) assay

Full-length coding sequences without stop codons of *CsTFL1*, *CsFT*, *CsFD*, *CsFDP*, *CsNOT2a*, *Cs14-3-3-3* and *Cs14-3-3-5* were amplified by PCR using gene-specific primers and introduced into pSPYNE-35S and pSPYCE-35S vectors containing the N or C terminus of YFP to construct in-frame fusion proteins (Walter et al., 2004). Before being transformed into the *Agrobacterium tumefaciens* strain GV3101, all constructs were confirmed by sequencing. The two plasmids were co-transformed into the abaxial side of 5- to 6-week-old tobacco (*Nicotiana benthamiana*) leaves to identify protein interactions (Ding et al., 2015b). The YFP signals were detected after 48 h co-infiltration using a Zeiss LSM 510 Meta confocal laser under 488 nm excitation wavelength. The primers used for BiFC are listed in Table S4.

## Supplementary Material

Supplementary information
