# Supplementary material for: CsTFL1 inhibits determinate growth and terminal flower formation through interaction with CsNOT2a in cucumber
Source: Development. 2019 Jul 29;146(14):dev180166. doi: 10.1242/dev.180166 (PMC6679365; doi:10.1242/dev.180166)
Supplement: Supplementary information [file develop-146-180166-s1.pdf]

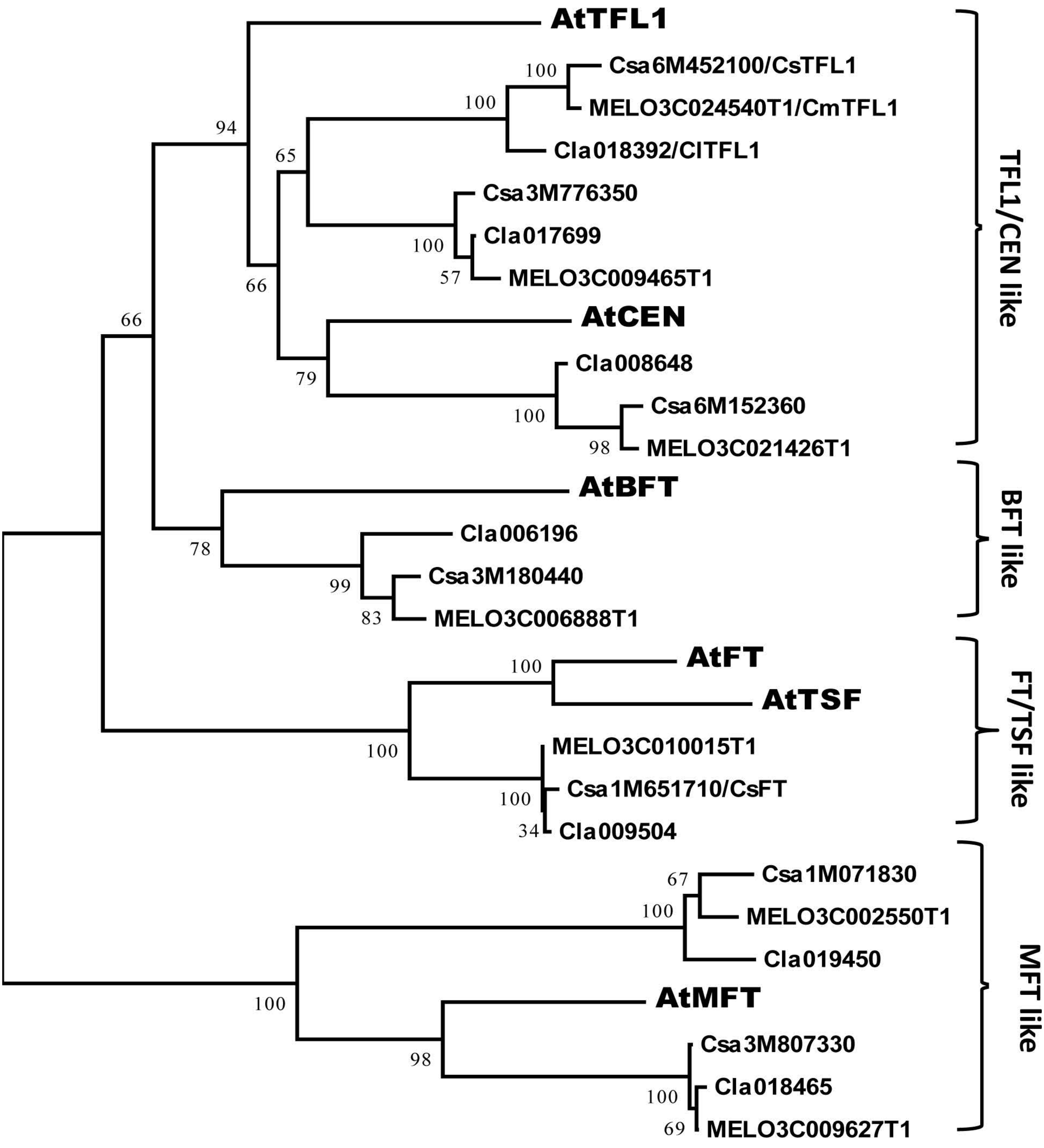

Fig. S1. Phylogenetic analysis of cucurbits PEBP genes. Phylogram of 27 PEBP family proteins from cucumber (Csa), melon (MELO), watermelon (Cla) and Arabidopsis (At). The scale bar represents 0.1 substitutions per site, and the numbers next to nodes are bootstrap values from 1,000 replicates.

Development • Supplementary information

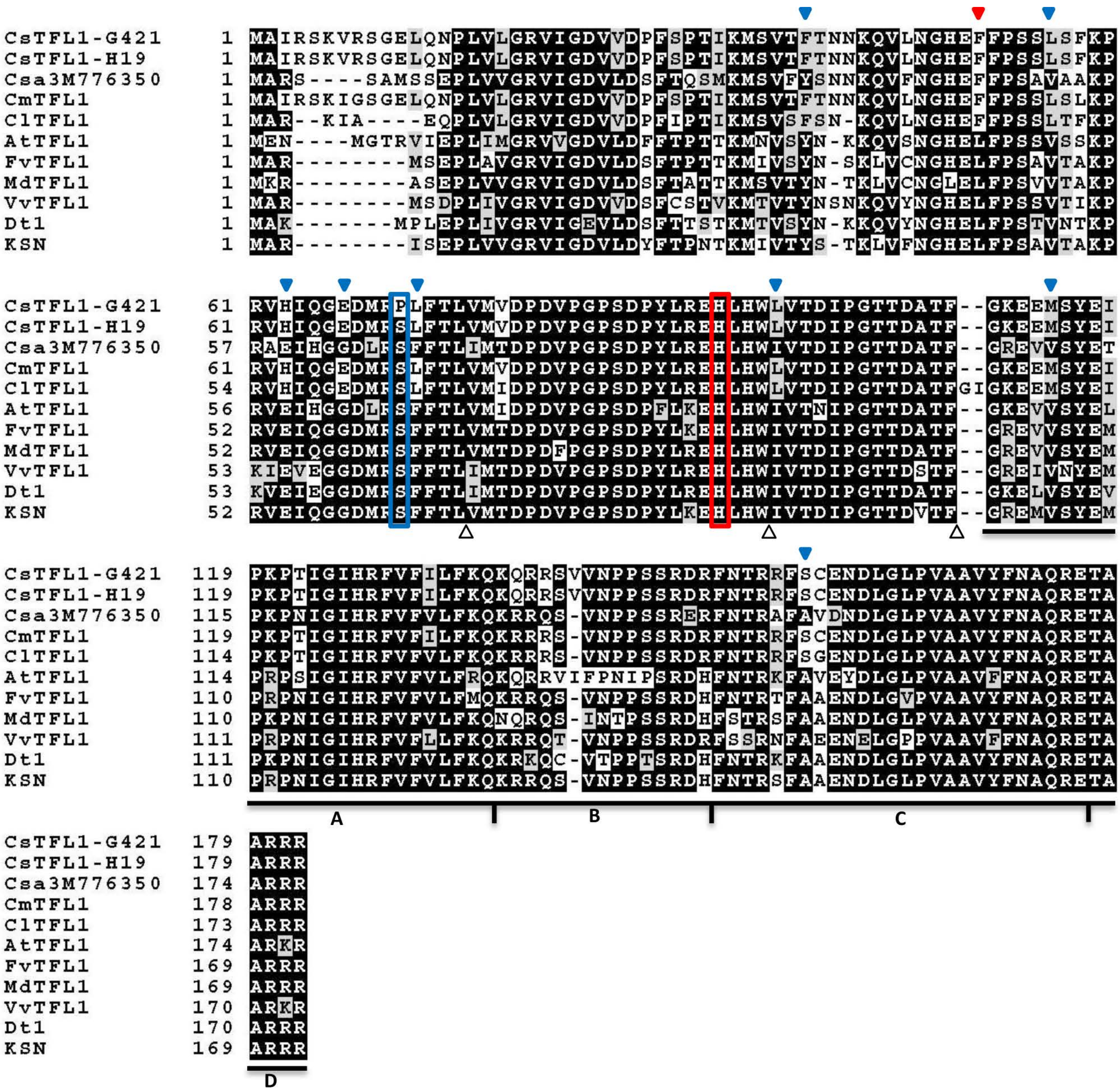

Fig. S2. Multiple alignments of CsTFL1 and TFL1-like genes in other species. Thin blue rectangle represents the SNP of S71P in G421. Thin red rectangle represents the conservative histidine in TFL1. Blue triangles represent the unconserved residues between Csa3M776350 and other cucurbits TFL1s. Red triangle represents the conserved residue in the cucurbit TFL1s. Open triangles represent the exon boundaries of TFL1. Four segments in the fourth exon of the TFL1s were shown as A, B, C and D. *At, Arabidopsis thaliana; Cs, Cucumis sativus; Cm, Cucumis melo L; Cl, Citrullus lanatus; Fv, Fragaria vesca; Md, Malus domestica; Vv, Vitis vinifera; Dt1 is from Glycine max; KSN is from Rosa chinensis.*

Development • Supplementary information

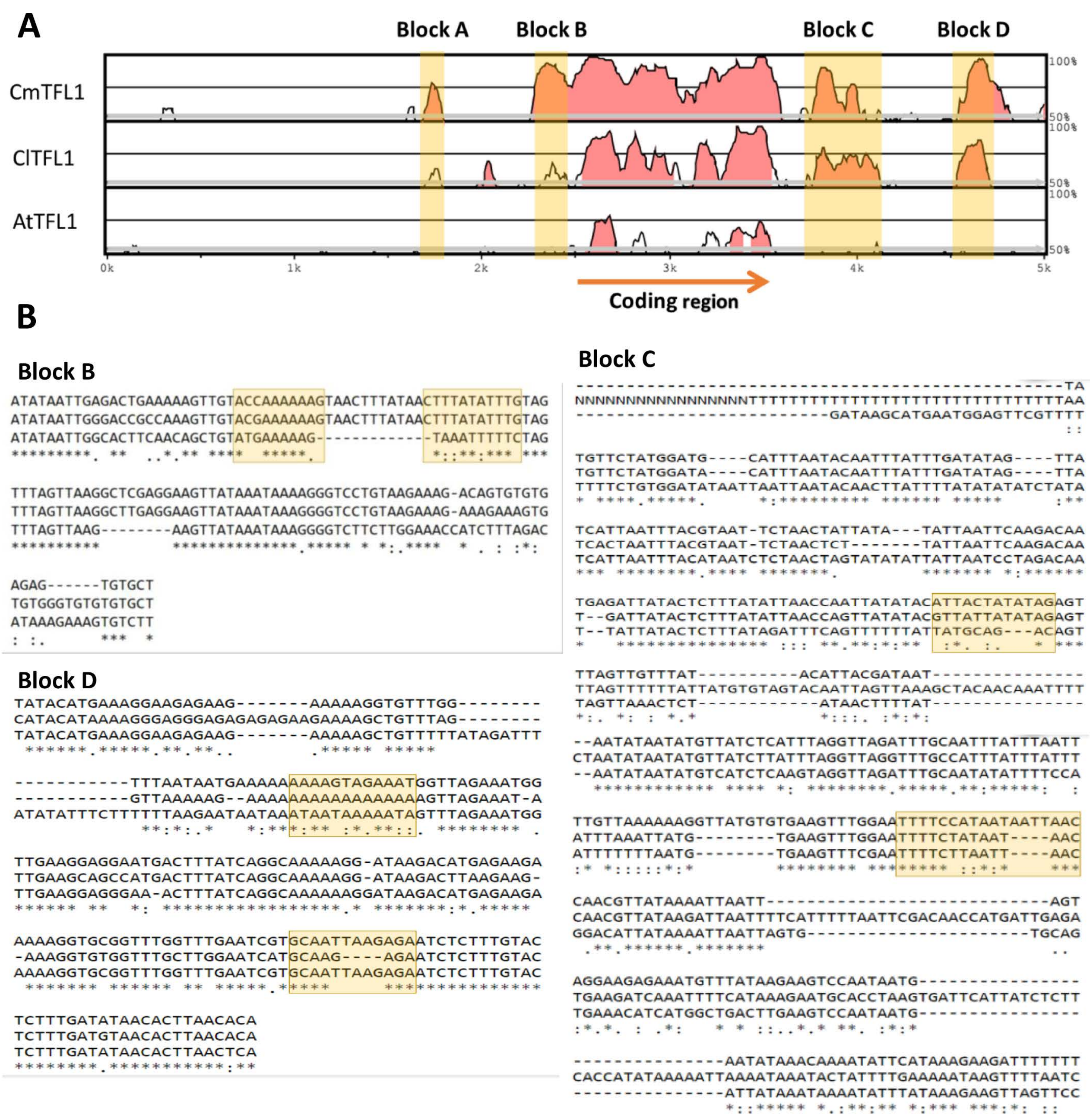

Fig. S3. Comparative analysis of the cucurbits TFL1s.(A) VISTA pairwise alignments of 5' and 3' region of melon Cm*TFL1*, watermelon *ClTFL1*, and Arabidopsis *AtTFL1* against cucumber *CsTFL1*. Graphical output shows the base pair identity (50-100% range) in a sliding window of 100 bp. Regions with >50% similarity are in red and the four conserved blocks outside the coding region (indicated by the arrow) are highlighted in orange.**(B)** The sequence of four identified conserved blocks in the 5' and 3' region of the *CsTFL1*, *CmTFL1* and *ClTFL1* were aligned using the GenomeNet online ClusterW server (http://www.genome.jp/tools-bin/clustalw). Six potential MADS-box binding sites (CArG box) were identified by searching against the JASPAR database and highlighted in orange.

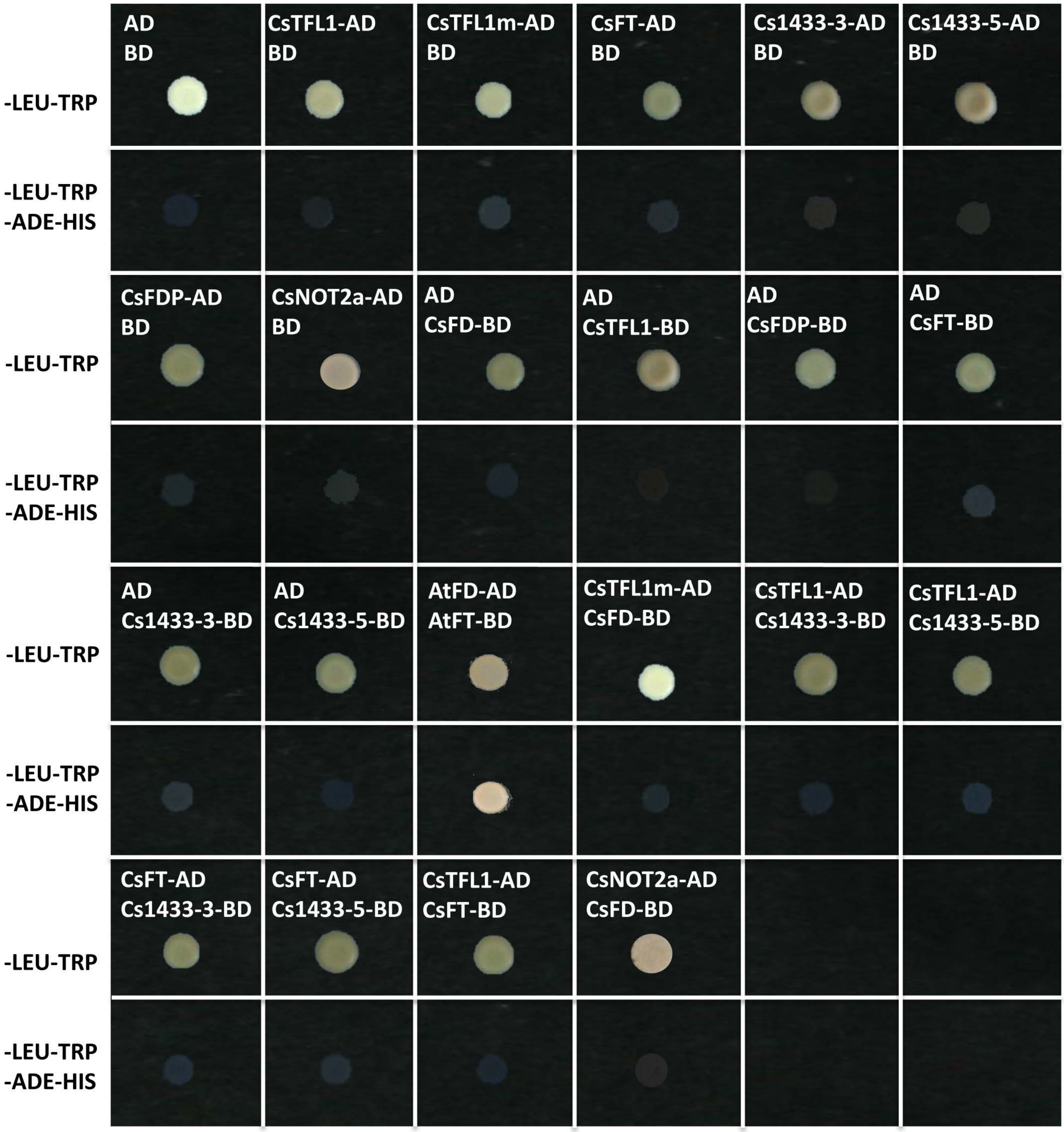

Fig. S4. Protein interactions as detected by yeast two-hybrid assays. The combination of empty vector pGBKT7 or pGADT7 was used as a negative control.

Development • Supplementary information

Table S1. Analysis of determinate traits in parental lines and segregation populations derived from G421 and H19

Year	Generation	Plants Tested	D： d Observed	D： d Ratio Tested	X^{2a}
	H19	30	30:0	D	
3 years	G421	28	0:28	d	
	F_1 H19×G421	42	42:0	D	
2010	RIL-F_8 H19×G421	139	72:67	1:1	0.18
2011	F_2 H19×G421	946	722:224	3:1	0.88

Table S2. Markers and genotype information used in this study.

Click here to Download Table S2

Table S3. Gene information used in this study

Gene name	Species	Gene ID in TAIR/ Cucurbit Database/ Genebank
AtTFL1	Arabidopsis	At5G03840
AtFT	Arabidopsis	At1G65480
AtBFT	Arabidopsis	At5G62040
AtMFT	Arabidopsis	At1G18100
AtCEN	Arabidopsis	At2G27550
AtTSF	Arabidopsis	At4G20370
AtFD	Arabidopsis	AT4G35900
AtHAN	Arabidopsis	AT3G50870
AtIND	Arabidopsis	AT4G00120
AtSPT	Arabidopsis	AT4G36930
CsFD	Cucumber	Csa3G002610
CsFDP	Cucumber	Csa6G051480
Cs1433-3	Cucumber	Csa2G369070
Cs1433-5	Cucumber	Csa4G094520
CsNOT21	Cucumber	Csa6G302150
CsLFY	Cucumber	Csa1G000050
CsSOC1	Cucumber	Csa6G076720
CsTEN	Cucumber	Csa5G644520
FvTFL1	Fragaria vesca	JN172097
MdTFL1	Malus x domestica	AB162040.1
VvTFL1	Vitis vinifera	AF378127
Dt1	Glycine max	ADF30943.1
KSN	Rosa	ADO64261.1

Development • Supplementary information

Table S4. Primer information used in this study

**Primers for genetic mapping and cloning**	
SSR01698-F	ACGATGAATTTTGCCGTAGC
SSR01698-R	GAAGAATCACTGAACCCCGA
SSR14859-F	CAAACCTAGCCTTAACCTTTGG
SSR14859-R	AAACCTTTTTGACGTTGAAGGA
SSR13251-F	GGTCAATCCAAAAGAGAAAGCA
SSR13251-R	ATCAACACCATTGACGACCA
SSR06632-F	TCAGATGTTGATTGGCTCTCA
SSR06632-R	AGGGCCAACATTAAAGGGTC
SSR01101-F	TGTGACCACTCATTACACCCA
SSR01101-R	TTTATTGTTTGGCCCATTCC
SSR06653-F	AAAGTTGGAAGGGTTGTGGTT
SSR06653-R	CTCTCTCGCGGATGTTTTGT
UW084417-F	GGAAGAAACAAACCTGCTG
UW084417-R	TAGTGGGTGGAGGTGAAAGG
UW085354-F	TGGAGCATATAAGTAAGGTTTAGGG
UW085354-R	TGGGCAAATAGTCATATTTTGAT
SSR10449-F	CACAATTTCCTCCGCTGTTT
SSR10449-R	CCTTTTCAGCTTCTTCTAATTTCTC
UW085356-F	GTGGAGATAGGAATTATTGGAGAG
UW085356-R	TGAATACTTGATTTATCCAGGCTGT
SSR17604-F	TTGAAGTGGGAGAGGAAGGA
SSR17604-R	TTCATTTTGATATTGAAGTTAACACG
UW085412-F	ACGATCCATATGCTTGTGTTT
UW085412-R	GATTCCAACGCACGTTTTCT
UW085076-F	GTCCCCAACATCCTTTCCTT
UW085076-R	TCATCGGTCAACCAATCAAT
SSR14934-F	ATACCTTGCCGGAGCCTTAT
SSR14934-R	ACTTTTTCAGCAGAGCAGCC
SSR15482-F	AAAAACAGCAGGGCAGAAGA
SSR15482-R	GTTGAGCTGTCTATGGGGGA
UW015248-F	CCCGAAATTTTTAGTTGGTCC
UW015248-R	CTTGAAATTCCTTACCCCACA
UW015253-F	GACAAAATTCAGAGTACAACACACC
UW015253-R	AAATGAAATGGATGATTTAGATTGA
TFL1-SNP-F	GGGTTCATATTCAAGGAGAAGATATGATATCATTGT
TFL1-SNP-R	GGAAATGGGTTTTCTCAAAGTTC
**Primers for gene amplification and vector construction**	
CsTFL1-clone-F	ATGGCAATTAGATCAAAAGTAAGAT
CsTFL1-clone-R	TTATCGCCTCCTTGCAGCA
CsTFL1-OE-F	GCTCTAGAATGGCAATTAGATCAAAAGTAAG
CsTFL1-OE-R	CCCCCCGGGTTATCGCCTCCTTGCAGC

Development • Supplementary information

CsTFL1-Sense-F	GGACTAGTCCATGGCAATTAGATCAAAAGTAA
CsTFL1-Sense-R	CGGGATCCCGCAATGGTCTCATATCTTCTCCT
CsTFL1-Antisense-F	TTGGCGCGCCAAATGGCAATTAGATCAAAAGTAA
CsTFL1-Antisense-R	CCATTTAAATGGCAATGGTCTCATATCTTCTCCT
CsTFL1-GFP-F	GGACTAGTATGGCAATTAGATCAAAAGTAAG
CsTFL1-GFP-R	TCCCCCGGGTCGCCTCCTTGCAGC
**Primers for qRT-PCR**	
CsTFL1-Q-F	GCGTCGGTCAGTAGTGAATCCTCC
CsTFL1-Q-R	TTATCGCCTCCTTGCAGCAGTTT
CsFT-Q-F	AATCAACCAAGAGTCGAGATTGG
CsFT-Q-R	TTGCACCTGTTGTAGCTGGAATA
Csa3M776350-Q-F	AGCAATGTCCTCAGAACCTCTTG
Csa3M776350-Q-R	AGCAACAGCAGAAGGAAAGAACT
CsUBIeq-Q-F	CACCAAGCCCAAGAAGATC
CsUBI-eq-Q-R	TAAACCTAATCACCACCAGC
ACTIN2-F	CCTTCGTCTTGATCTTGCGG
ACTIN2-R	AGCGATGGCTGGAACAGAAC
**Primers for in situ probes**	
CsTFL1-T7	tgTAATACGACTCACTATAGGGCAATGGTCTCATATCTTCTCCT
CsTFL1-Sp6	GATTTAGGTGACACTATAGaatGCTATGGCAATTAGATCAAAAGTAA
**Primers for yeast two-hybrid, BiFC and pull-dowm**	
AtHAN-Y2H-F	GGAATTCCATATGATGATGCAGACTCCGTACACT
AtHAN -Y2H-R	CGGGATCCCTCTGGTAAAGTCATGGACAAGAC
AtFT-Y2H-F	CGGAATTCATGTCTATAAATATAAGAGACCCTCTTATAGTAAGC
AtFT -Y2H-R	CGGGATCCAAGTCTTCTTCCTCCGCAG
AtFD-Y2H-F	CGGAATTCATGTTGTCATCAGCTAAGCATCAG
AtFD-Y2H-R	CGGGATCCAAATGGAGCTGTGGAAGACCG
CsTFL1-Y2H-F	GGAATTCCATATGATGGCAATTAGATCAAAAGTAA
CsTFL1-Y2H-R	CGGGATCCCTTATCGCCTCCTTGCAGCAGT
CsFT-Y2H-F	GGAATTCCATATGATGCCAAGAGATCGTGACCC
CsFT-Y2H-R	CGGAATTCTTAATAATCATCTTGGACTCTTCT
CsFD-Y2H-F	GGAATTCCATATGATGGAAGAAGTTTGGAAAGACATAAG
CsFD-Y2H-R	CGGGATCCCCTAAAACGGCGCCGTTGATG
Cs1433-3-Y2H-F	GGAATTCCATATGATGGCTGCCGCTCCTTCTGT
Cs1433-3 -Y2H-R	CCCCCCGGGCTGCTTTTCATCCTCACGCTT
Cs1433-5-Y2H-F	GGAATTCCATATGATGTCGCCTGCTGATTCTTCA
Cs1433-5-Y2H-R	CCCCCCGGGCTGTCCATGTCCCTCCCCTGA
CsFDP-Y2H-F	CGGAATTCATGGGGATTCAAACTATGGGGTC
CsFDP-Y2H-R	CGGGATCCGAACGGCGCTGACGATGTTCTTCG
CsNOT2a-Y2H-F	CGGAATTCATGTCGGGTTTACTTAATTCATCTC
CsNOT2a-Y2H-R	CGGGATCCATGTTGCGATAGAACTGGTCTC
AtIND-BiFC-F	TGCTCTAGAATGGAAAATGGTATGTATAAAAAG
AtIND-BiFC-R	CGCGGATCCGGGTTGGGAGTTGTGGTA

AtSPT-BiFC-F	TGCTCTAGAATGATATCACAGAGAGAAGAAAGA
AtSPT-BiFC-R	CGCGGATCCAGTAATTCGATCTTTTAGGTCAG
CsFT-BiFC-F	GGACTAGTATGCCAAGAGATCGTGACCC
CsFT-BiFC-R	CCCCCCGGGATAATCATCTTGGACTCTTCTCC
CsFD-BiFC-F	GGACTAGTATGGAAGAAGTTTGGAAAGACATAAG
CsFD-BiFC-R	CCCCCCGGGAAACGGCGCCGTTGATG
Cs1433-3-BiFC-F	GGACTAGTATGGCTGCCGCTCCTTCTGT
Cs1433-3 -BiFC-R	CCCCCCGGGCTGCTTTTCATCCTCACGCTT
CsFDP-BiFC-F	CGGGATCCATGGGGATTCAAACTATGGGGTC
CsFDP-BiFC-R	CCCTCGAGGAACGGCGCTGACGATGTTCTTCG
CsNOT2a-BiFC-F	CGGGATCCATGTCGGGTTTACTTAATTCATCTC
CsNOT2a-BiFC-R	CCCTCGAGATGTTGCGATAGAACTGGTCTC

Development • Supplementary information
